# Synchronous Triple Malignancies in an Indian Albino: A Case Report

**DOI:** 10.7759/cureus.3190

**Published:** 2018-08-23

**Authors:** Danny Darlington, Susrutha Puthanmadhom Narayanan, Fatima Shirly Anitha

**Affiliations:** 1 Urology, Government Stanley Medical College And Hospital, Chennai, IND; 2 Bachelor of Medicine Bachelor of Surgery, Jawaharlal Institute of Postgraduate Medical Education and Research (JIPMER), Pondicherry, IND; 3 Pediatrics, Church of South India Kalyani Multispeciality Hospital, Chennai , IND

**Keywords:** basal cell carcinoma, melanoma, oculocutaneous albinism, squamous cell carcinoma

## Abstract

Oculocutaneous albinism (OCA) is a heterogenous disorder of skin pigmentation characterized by hypopigmentation of the skin, hair, and eyes. The absence of melanin predisposes these individuals to ultraviolet rays induced malignancies. Basal cell carcinoma (BCC) and squamous cell carcinoma (SCC) in OCA have been rarely reported. Malignant melanoma (MM) of the skin is also very rarely reported. Synchronous BCC, SCC, and MM are exceedingly rare. We report one such case managed successfully with surgical treatment. All the three malignancies were localized cancers and hence the outcome was good. The importance of regular follow up and periodic self-examination in such predisposed individuals are highlighted.

## Introduction

Albinism is a disorder of pigmentation characterized by a generalized reduction in melanin pigment or the absence of it in the skin, hair, and eyes. Its prevalence is estimated to be one in 20,000 individuals worldwide. Oculocutaneous albinism (OCA) is caused by a heterogeneous group of mutations involving melanin synthesis. Mutations in tyrosinase (TYR) gene (11q14-q21), resulting in impaired tyrosinase activity, are by far the most common and affect up to 50% of cases worldwide [1-2]. Ocular features associated with albinism include nystagmus, photophobia, foveal hypoplasia, and decreased visual acuity. Lack of melanin in the integument predisposes these individuals to a multitude of cutaneous malignancies as well. Actinic keratosis progressing to squamous cell carcinoma (SCC) is the most frequently reported skin cancer in albinos. The risk for basal cell carcinomas (BCC) is also higher in these individuals as compared to the general population [3]. Among all the skin cancers reported in the albino population, malignant melanomas (MM) are extremely rare [4]. Very few cases have been reported in the literature and majority of the reported cases have been amelanotic [5]. Here, we report the case of an Indian albino with concurrent melanotic MM, SCC, and BCC in the head and neck region.

## Case presentation

**Figure 1:**
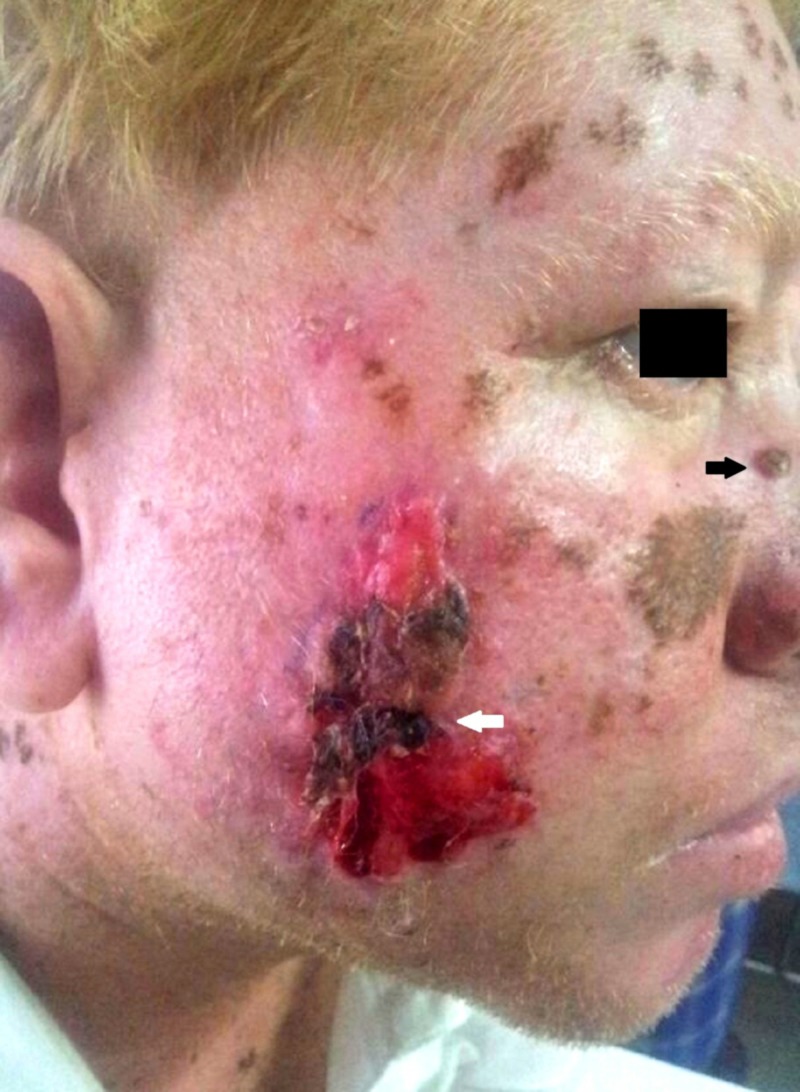
Clinical photograph of the patient showing basal cell carcinoma of the nose (black arrow) and malignant melanoma of the right cheek which is characteristically melanotic (white arrow)

**Figure 2:**
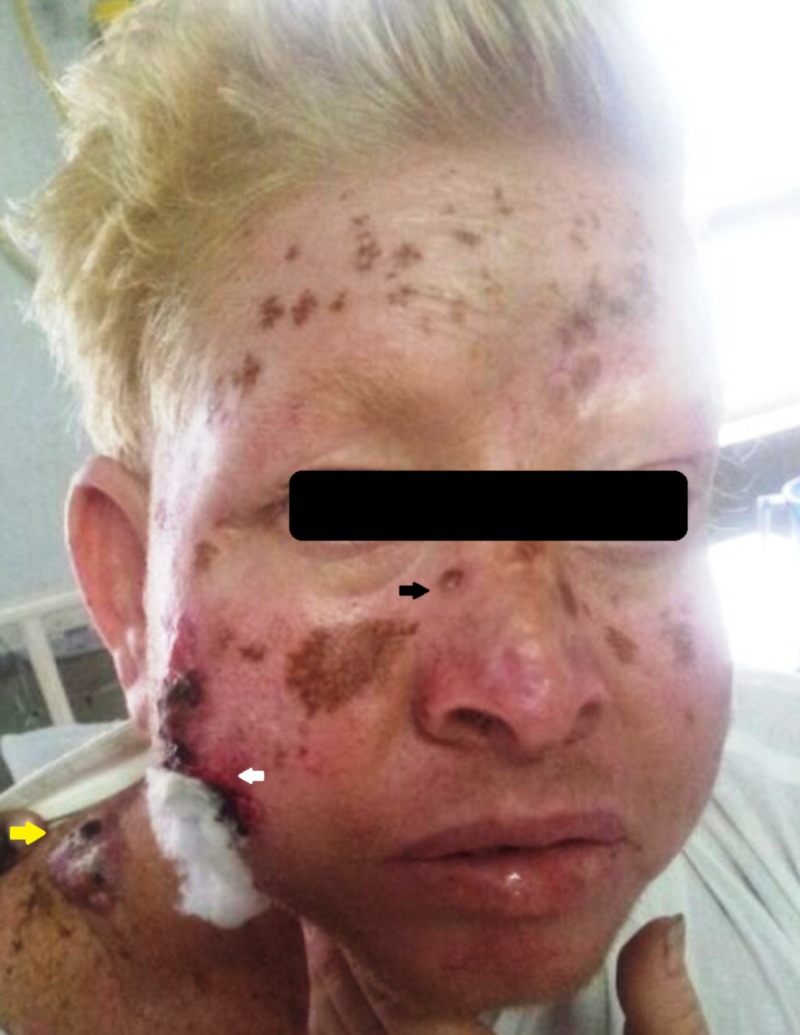
Clinical photograph depicting the basal cell carcinoma (black arrow), malignant melanoma (white arrow), squamous cell carcinoma of the right shoulder (yellow arrow), and multiple melanotic nevi involving the face

**Figure 3:**
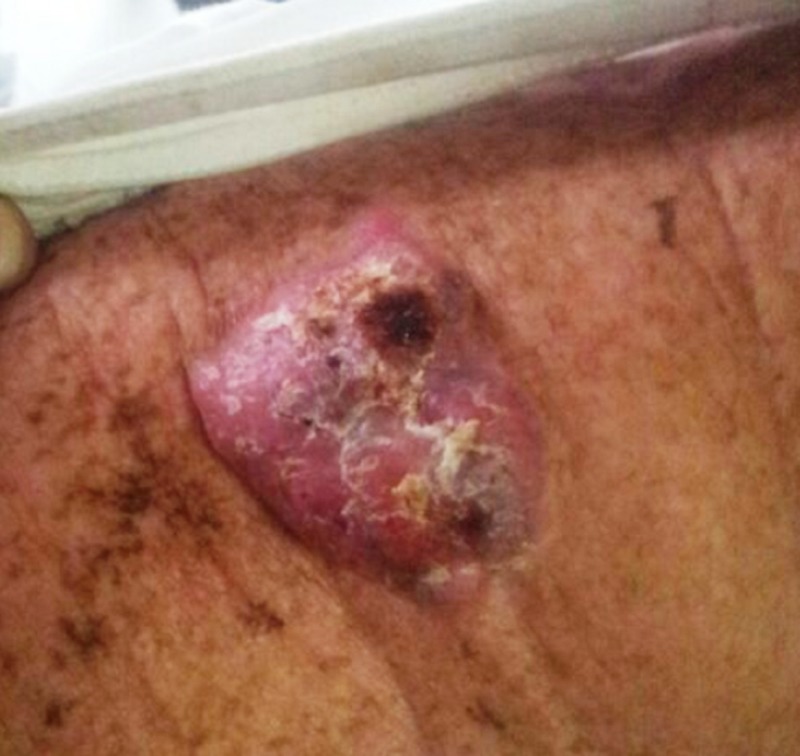
Clinical photograph of squamous cell carcinoma over the right shoulder

A 40-year-old Indian farmer, with a prior diagnosis of OCA, presented to the surgical outpatient department of our tertiary care hospital with complaints of a rapidly growing dark-colored ulcer on his right cheek and a smaller ulcer on his right shoulder that was noticed recently. He had a history of multiple dark, flat, patches on the skin since childhood. The patches on the right cheek progressed into a hyperpigmented, raised ulcer. Personal and family histories were noncontributory. On examination, there was an irregular, pigmented, raised, crusted, and indurated ulcer on the right cheek measuring 5 cm × 3 cm suggestive of melanotic MM. There was a 5 mm round pearly nodule, suspicious of BCC, located lateral to the nasal bridge on the right side. There were multiple benign-appearing pigmented macules distributed all over his body and face. The lesion on the right shoulder measured 3 cm × 2 cm. It was ulcerated and had a heaped up appearance with a necrotic base, suggestive of SCC (Figures 1-3). There were no palpable lymph nodes in the neck or preauricular region. There was no clinical evidence of systemic metastasis.

**Figure 4:**
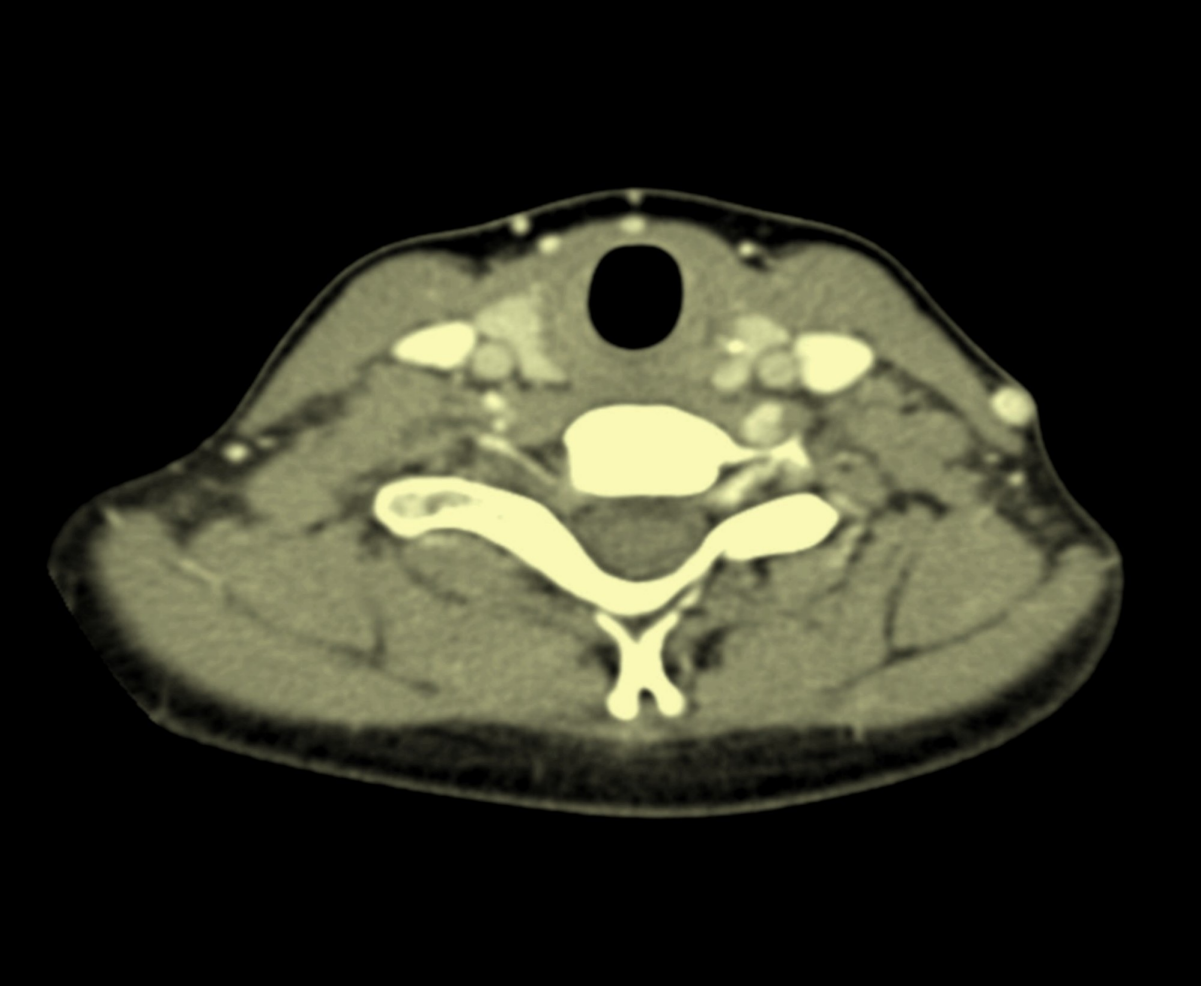
Contrast-enhanced computed tomography (CT) of the neck showing the absence of cervical metastases

**Figure 5:**
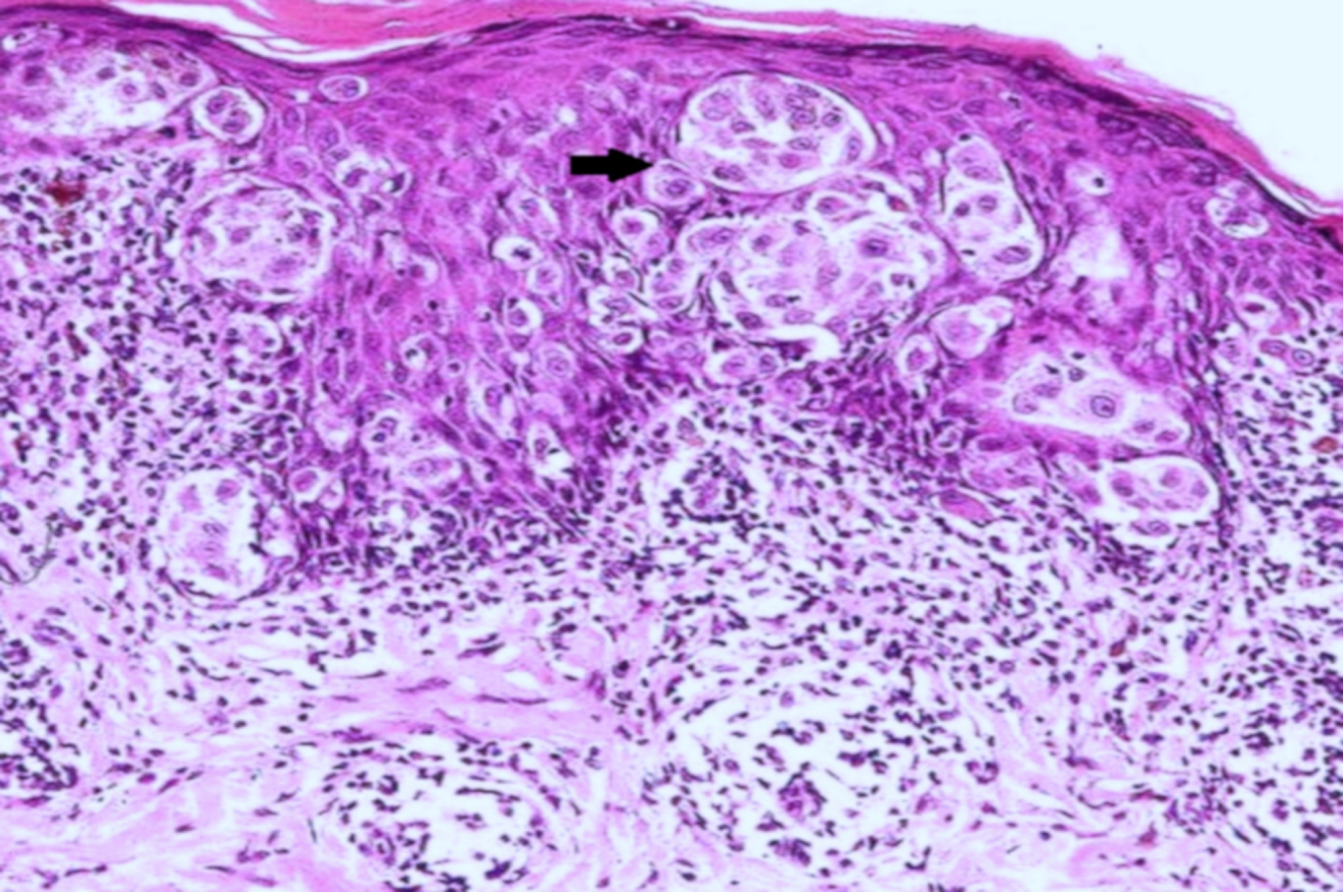
Histopathological image of malignant melanoma depicting nests of atypical melanocytes (arrow) invading the papillary dermis (hematoxylin and eosin stain, 200x magnification)

**Figure 6:**
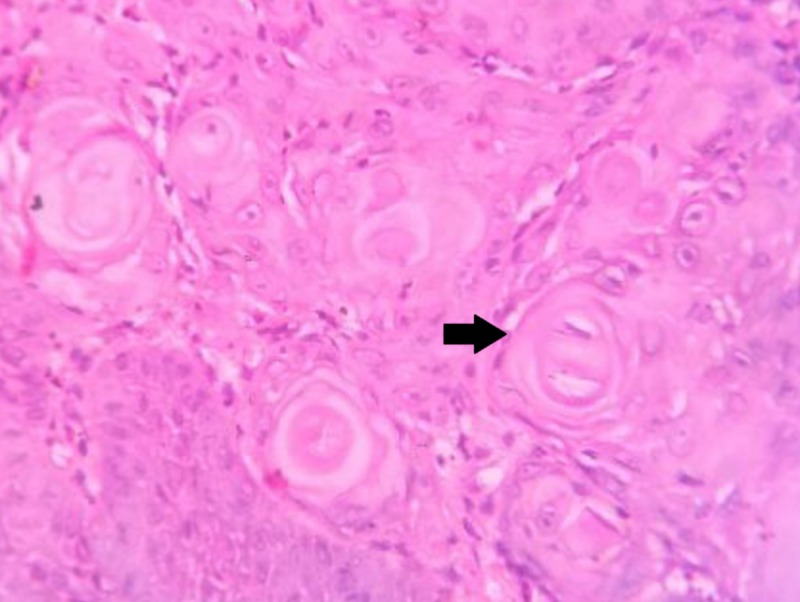
Histopathological image of squamous cell carcinoma demonstrating keratin pearls marked by the black arrow (hematoxylin and eosin stain, 40x magnification)

**Figure 7:**
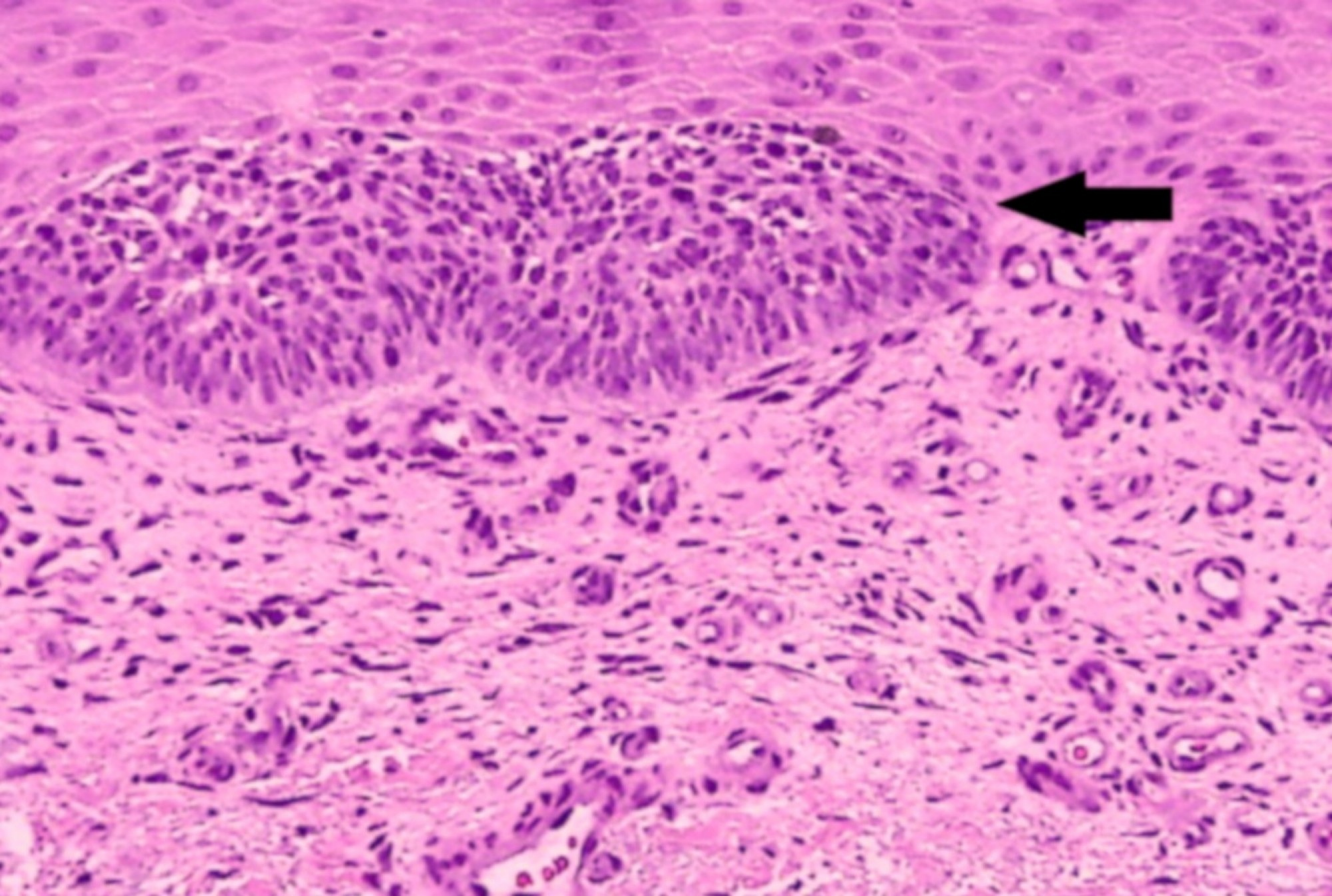
Histopathological image of basal cell carcinoma illustrating basaloid cells (arrow) with hyperchromatic nuclei and characteristic peripheral palisading (hematoxylin and eosin stain, 40x magnification)

Hemogram and renal function tests were unremarkable. He had a normal chest roentgenogram, computed tomography (CT) of the neck, and ultrasonogram of the abdomen, thus ruling out metastatic disease (Figure 4). Due to a high clinical suspicion for malignancy, an incisional biopsy was not performed preoperatively for any of the three lesions. Wide local excision of all the three lesions was performed under general anesthesia and the excisional biopsy samples were sent for histopathological assessment. Primary closure was performed in the right shoulder following excision. Excision sites on the face were closed by rotational flap repair. The lesion on the right cheek was confirmed to be superficial spreading MM, the one on the shoulder was diagnosed as well-differentiated SCC, and the lesion on the nose was found to be nodular BCC on histopathological examination. The MM had a Breslow thickness of 0.7 mm and was staged as Clark’s level 2 (Figure 5). The SCC was staged T2N0M0 and BCC was staged T1N0M0 (Figures 6-7). Margins of all the excised tissues were free of malignancy. He had an unremarkable postoperative course in the hospital. At the time of discharge, surgical wounds and flaps were healthy and healing. He was advised on the need for sun protection and close surveillance.

## Discussion

OCA is an autosomal recessive genetic disorder that could be associated with mutations involving a heterogeneous group of genes. It occurs with a prevalence of 1 in 20,000 worldwide. When OCA occurs as a nonsyndromic disorder, specific genes get mutated such as TYR, OCA type 2 (OCA2) (P protein gene), tyrosinase-related protein 1 gene (TYRP1), and SLC45A2 protein gene (SLC45A2), that are necessary for melanin biosynthesis [6]. OCA1 involves the TYR gene that codes for tyrosinase and is the most common mutation implicated globally as well as among Caucasians and Indians [1-2]. OCA type 2 (OCA2), with a mutated P gene, is more common in the African population [1,7]. It may also occur as part of Hermansky-Pudlak syndrome along with platelet abnormalities and ceroid lipofuscin storage abnormalities [3]. Clinically OCA causes hypopigmentation of the eye, skin, and hair. Ocular manifestations include nystagmus, strabismus, iris and retinal hypopigmentation, reduced visual acuity, and photophobia [3]. Patients with OCA1 present with white skin and hair, blue to pink translucent irides, amelanotic nevi, poor visual acuity, and severe photophobia due to a total absence of melanin. The other variants present with less severe symptoms and usually accumulate melanin over time. Pigmented skin lesions such as nevi and freckles are relatively more common in OCA2. It is associated with a better visual acuity than OCA1. Individuals with OCA are at a higher risk for developing skin malignancies due to lack of protection from ultraviolet radiations. Physical development, intelligence quotient, reproductive health, and longevity are not affected by the disease [3]. The patient described above had mildly pigmented skin and hair, blue to grey iris and pigmented skin lesions. Although his vision was not severely impaired, he had severe photophobia. This picture fits with the OCA2 phenotype, but genetic testing was not feasible considering his economic status.

Ultraviolet radiations, predominantly ultraviolet B, induce carcinogenic pyrimidine dimerization mutations in deoxyribonucleic acid (DNA) that are reported in over 50% of cases of SCC and BCC. In pigmented skin, melanosomes that contain melanin form supranuclear caps inside keratinocytes. Melanin acts as a barrier and absorbs most of the ultraviolet radiations that fall on the skin, thus protecting the underlying nuclear DNA [8]. In OCA, melanin synthesis is impaired and consequently, patients are at an increased risk for development of skin malignancies. SCC is the most common skin malignancy seen in OCA, followed by BCC [4]. On the other hand, melanomas occur rarely in OCA and the majority of those that have been reported are amelanotic [5]. There are few case reports that have described the synchronous occurrence of more than one type of skin malignancy or premalignant lesion in individuals with OCA. Chatterjee et al. reported the case of an Indian albino who presented with concurrent BCC and actinic keratosis, a precursor of SCC [9]. A prospective study by van der Westhuizen et al. described 16 OCA patients with melanocytic or pigmented skin lesions. Ten patients out of them had concurrent or prior nonmelanoma skin cancers [10]. Here, we report the case of a patient with a prior diagnosis of OCA, presenting with a grossly pigmented malignant skin lesion which turned out to be melanotic superficial spreading melanoma histologically. He also had synchronous SCC and nodular BCC which is an extremely rare presentation that merits attention.

On imaging, there was no evidence of lymph node or systemic metastasis. Underlying OCA is not known to alter the management of skin malignancies. This is evident from various case reports where nonmetastatic skin tumors were managed with wide local excision in patients with albinism [9,11]. Hence, each individual lesion was excised and submitted for histological assessment. In the face, rotational flap cover was done for the surgical defects while the surgical wound over the right shoulder was closed primarily. Follow up is uneventful.

## Conclusions

This case is reported for its rarity. It depicts the rare occurrence of concomitant melanotic melanoma and nonmelanoma skin cancers in OCA. All albinos presenting with cutaneous malignancies must be subjected to extensive evaluation for other malignancies as well. Surgeons and dermatologists should be aware of this possibility to prevent delays in diagnosis and treatment.
